# Intra-articular therapy with methotrexate or tumor necrosis factor inhibitors in rheumatoid arthritis: a systematic review

**DOI:** 10.1186/s12891-021-04651-5

**Published:** 2021-09-15

**Authors:** Megan M. Sullivan, Michael M. Pham, Lisa A. Marks, Fawad Aslam

**Affiliations:** 1 Division of Rheumatology, Department of Internal Medicine Mayo Clinic, 4500 San Pablo Road, Jacksonville, FL 32224 USA; 2 Division of Rheumatology, Department of Internal Medicine Mayo Clinic, Scottsdale, AZ USA; 3 Mayo Clinic Libraries Arizona, Mayo Clinic, Scottsdale, AZ USA

**Keywords:** Rheumatoid arthritis, Intraarticular, Monoarthritis, Joint, Injection, Corticosteroid, Methotrexate, Review, Tumor necrosis factor inhibitor

## Abstract

**Background:**

Persistent monoarthritis in otherwise well-controlled rheumatoid arthritis presents a therapeutic challenge. Intra-articular (IA) steroids are a mainstay of treatment, though some have queried whether IA disease modifying anti-rheumatic drugs (DMARD) and biologics can be used in those who fail steroid injections.

**Methods:**

A systematic literature review was conducted using four medical databases to identify randomized, controlled trials assessing IA therapies in RA patients. Included studies underwent Cochrane Risk of Bias 2 assessment for quality.

**Results:**

Twelve studies were included, 6 of which examined intra-articular (IA) TNF inhibitors (TNFi), and 6 studies evaluating IA methotrexate. Of those evaluating IA TNFi, one study reported statistical improvement in TNFi therapy when compared with placebo. The remaining 5 studies compared IA TNFi therapy with steroid injections. IA TNFi had statistically improved symptom scores and clinical assessments comparable with IA steroid treatments.

In the 6 studies evaluating IA methotrexate, the addition of methotrexate to steroid intra-articular therapy was not found to be beneficial, and singular methotrexate injection was not superior to the control arms (saline or triamcinolone). Risk-of-bias (ROB) assessment with the Revised Cochrane ROB tool indicated that 2 of 6 TNFi studies were at some risk or high risk for bias, compared with 5 out of 6 methotrexate studies.

**Conclusion:**

For persistent monoarthritis in rheumatoid arthritis, IA methotrexate was not found to have clinical utility. Intra-articular TNFi therapy appears to have equal efficacy to IA steroids, though the optimal dose and frequency of injections is yet unknown.

**Supplementary Information:**

The online version contains supplementary material available at 10.1186/s12891-021-04651-5.

## Background

The continued expansion of immunosuppressive medication options has greatly improved disease activity control in rheumatoid arthritis (RA) patients. However, persistent symptomatic monoarthritis can be a problem in some patients who otherwise have satisfactory disease control [1]. Intra-articular (IA) steroid injections have remained a treatment cornerstone for these patients, but the need for other therapies is clear. Patients can fail such therapy, may develop adverse effects or have comorbidities that are exacerbated by intra-articular steroids. Almost 50% of patients can relapse after an IA corticosteroid injection [2]. There can be patient and provider hesitation, primarily driven by concern for adverse effects, to add or escalate systemic immunosuppressive treatment for isolated monoarthritis. A similar situation arises in some patients with isolated inflammatory monoarthritis, without an associated systemic disease or even an underlying diagnosis, where systematic immunosuppressive treatment may seem aggressive compared to the more localized IA therapy.

The aforementioned clinical scenarios are uncommon but real problems seen in everyday rheumatology clinical practice. After exhausting IA steroid treatments, therapeutic paths forward are unclear. In such situations, surgical and radiation-induced synovectomy have been employed as treatment approaches [3, 4]. Several studies have looked at the utility of other IA immunosuppressive treatments as a management option for persistent inflammatory monoarthritis. The medicines studied have included methotrexate and tumor-necrosis-factor inhibitors (TNF-i). The aim of this systematic review was to evaluate the suitability and effectiveness of IA methotrexate and/or IA TNF-i to treat persistent monoarthritis in RA patients.

## Materials and methods

### Eligibility criteria

This systematic review was conducted in accordance with the recommendations of Preferred Reporting Items for Systematic Reviews and Meta-analysis (PRISMA) [5]. Studies fulfilling the following criteria were included: 1) randomized, controlled trials with an objective of evaluating efficacy of novel (defined as disease modifying anti-rheumatic drugs [DMARD] e.g. methotrexate and/or biologics e.g. etanercept) IA agents against standard of care IA agents e.g. corticosteroids, 2) studies included patients with rheumatoid arthritis and/or persistent inflammatory arthritis, 3) studies enrolled human subjects.

### Search strategy and information sources

A literature search was conducted on 10th August 2020 by a trained, experienced medical librarian utilizing medical subject heading (MeSH) and text words related to the study question. The following keywords and their combinations were used in the search strategy to identify various DMARD IA therapies that may have been described in the literature: “intra-articular joint injection”; “intra-articular injection”; “rheumatoid arthritis”; “arthritis”; “antirheumatic agents”; “anti-inflammatory agents”; “immunosuppressive agents”; “abatacept”; “monoclonal antibodies”; “belimumab”; “certolizumab pegol”; “certolizumab”; “cyclophosphamide”; “cyclosporine”; “etanercept”; “leflunomide”; “methotrexate”; “sirolimus”; “adalimumab”; “enbrel”; and “infliximab”. The following MeSH terms were used in conjunction with their keyword counterparts. Keywords and MeSH terms were combined using the Boolean operators “AND” and “OR”. A sample search strategy is given as supplementary Table 1.

Searched databases included Ovid MEDLINE, Ovid EMBASE, Scopus, and Web of Science. There was no publication date limit. Search was restricted to English language studies. Non randomized studies, studies with non-adult population studies and non-English studies were excluded. Conference abstracts were not excluded. Bibliography of identified studies was scanned to identify further studies for inclusion. Additional relevant studies identified from review articles on topics of intra-articular joint injections and rheumatoid arthritis therapy were also included. Per the 2020 PRISMA checklist, a sample strategy is provided in the supplementary material.

### Study selection and data collection

Reference duplication assessment and data management were performed with EndNote library (version X9, Clarivate analytics). Two authors (FA and MM) independently sorted the identified abstracts for inclusion in the review. The full text article was reviewed for inclusion determination if an abstract was not available at this stage. The identified abstracts led to full-text review by each of the two authors for eligibility. Conflicts were resolved by consensus. Exclusions of full-text papers were recorded with their listed exclusion criteria. Information from the final included papers was reported by one reviewer (MM), and then reexamined by the second reviewer (FA) for accuracy. Data recorded included first author, publication year, study location, study design, study participant number, treatment arm regimens, outcome assessments, and adverse reactions to therapeutic interventions. Due to expected study heterogeneity, summary measures were not calculated

### Assessment of methodologic quality

The risk of bias in included studies was further evaluated by the Cochrane Risk of Bias 2 (RoB2) for randomized trials [6]. The RoB2 assesses risk of bias in five domains, including the randomization process, intended interventions, missing outcome data, measurement of outcome, and selection of the reported result. Each domain is rated as: high risk of bias, low risk of bias, or unclear risk of bias. Two authors (MM and MP) independently assessed the studies and resolved conflicts through consensus. In absence of consensus, an additional author (FA) gave the final assessment.

## Results

**Fig. 1:**
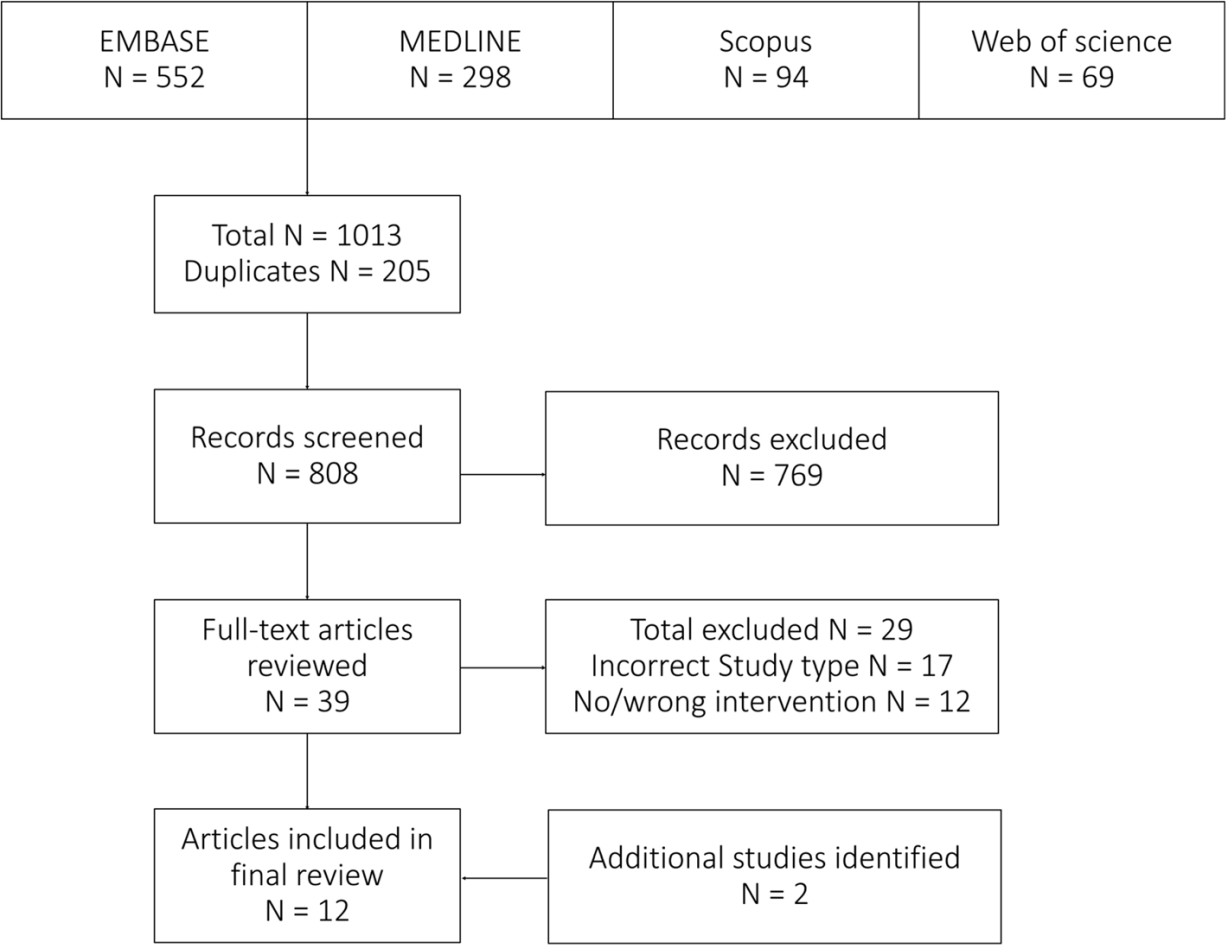
Study selection flowchart for systematic review

A total of 1013 citations were retrieved from the medical database searches. After removing duplicate references, 808 citations were reviewed based on inclusion and exclusion criteria. Full texts of 39 studies were reviewed, of which 29 were excluded:16 for the incorrect study type and 12 for an intervention other than IA DMARDs. Two additional studies were identified on bibliography review of the relevant literature. 12 studies were included in the final review. Study selection flowchart is shown in Fig. 1. Six studies investigated IA methotrexate while five studied IA etanercept. One study investigated different biologic agents: infliximab, etanercept, or adalimumab. Additional studies evaluating adalimumab did not meet our inclusion criteria.

### Intraarticular methotrexate

Table 1 gives the details of the six included studies. Five studies evaluated knees only and the only study not doing so, studied elbows, wrists and ankles. The included studies had variable methotrexate dosing (ranging from 5 mg to 80 mg), administration protocols, follow-up durations and outcome measures. Three of the six studies did not report on adverse effects.

**Table 1:** Randomized Control Trials Evaluating Intraarticular Methotrexate for Persistent Monoarthritis in RA

Author, Year, Country	N of RA	Objective (s)	Study Design	Time in Weeks	Primary Outcome	Joints	Results*	Conclusions	Side Effects
Marks [7], 1976, United Kingdom	12 unclear allocation distribution	Comparison of IA MTX + hydrocortisone vs hydrocortisone alone	Randomized, Single-blind	36	Pain and physician assessment. Not clearly specified.	Knee	5 patients in each group felt improvement following injection, 3 patients had objective improvement on knee examination in each group	MTX + hydrocortisone was not superior to hydrocortisone alone	No adverse events reported, though CBC and LFT’s had been evaluated
Bird [8], 1977, England	42 total, 23 with RA MTX: 9 Steroid: 14	Comparison of IA MTX with IA triamcinolone hexacetonide by thermography	Randomized	3	Thermography	Knee	The thermographic index improved in the triamcinolone group and was sustained through 3 weeks (0.02 > *p* > 0.01 at 7 and 14 days) when compared with MTX. More patients rated their pain as improved in the steroid group (*p* < 0.0005)	Triamcinolone was superior to MTX in reducing thermographic indices of injected knee joints	Not discussed
Hall [9], 1978, England	20 total, 15 with RA MTX: 3 Saline: 4 MTX & saline to one knee apiece: 8	Comparison of IA MTX vs Saline	Randomized, Double-blind	12	Clinical assessment; Arthroscopy findings on day 0 and after 12 weeks	Knee	Clinical measures improved in both groups, though there were not differences between groups. Less synovial inflammation was seen on 3-month arthroscopy regardless of treatment group	No benefit of MTX over saline	Not assessed
Blyth [10], 1998, Scotland	82 Steroid: 27 Steroid + MTX: 28 Steroid + Rifampicin: 27	Comparative study of IA triamcinolone, triamcinolone and rifampicin, and triamcinolone and MTX	Randomized, Single-blind	24	5-point pain scale	Knee	Triamcinolone + rifampicin resulted in statistically significant pain control at 3 months (*p* = 0.039), and the percentage of pain free patients was higher (*p* < 0.001). All groups improved compared to baseline, but no significant differences noted between triamcinolone +MTX to triamcinolone alone	Addition of MTX to triamcinolone did not provide any additional relief	11/28 patients had post-injection pain flares with rifampicin. 1 patient who received MTX had mouth ulcers 10 days after injection
Hasso [11], 2004, United Kingdom	38, 29 with RA MTX + steroid: 20 Steroid: 18	Comparison of IA MTX + triamcinolone vs triamcinolone alone in knee synovitis	Randomized, Double-blind	24	Patient and assessor global assessments of disease activity, knee pain VAS, duration of stiffness, joint circumference	Knee	Symptoms scores improved significantly in both groups with worsening between week 12–24, but no difference between treatment groups. 9 patients required repeat corticosteroid injections (5 in the triamcinolone group and 4 in the MTX group)	The addition of MTX to steroid injection did not improve symptom scores or clinical response compared with triamcinolone alone in chronic knee synovitis	11 patients had mild elevation of liver transaminases, did not clarify treatment group
Mortada [12], 2018, Egypt	100 MTX: 56 Steroid: 44	Comparison of IA MTX vs triamcinolone acetonide	Randomized, Single-blind	20	VAS, US findings	Ankle, wrist, and elbow	Clinical parameters and ultrasound findings improved in both groups by week 8. The clinical improvement continued in the MTX group to week 20, but plateaued in the steroid group (*p* = 0.04)	Repeated IA MTX injections resulted in a decrease of synovitis in medium-sized joints when compared with a single triamcinolone injection	2 participants in MTX group had oral ulcers, 1 had post-injection nausea. 3 in the steroid group had joint flares

*CBC* complete blood count; *LFT* liver function tests; *MTX* methotrexate; *N* number; *RA* rheumatoid arthritis; *US* ultrasound; *VAS* visual analog scale

*: When not given, *p*-value was not reported in the study or was statistically not significant

The first randomized trial compared IA methotrexate plus IA hydrocortisone with IA hydrocortisone alone in 12 RA patients with persistent knee synovitis [7]. No incremental benefit from IA methotrexate, based on clinical assessment at 3 months, was detected. They did not report on adverse effects. Then in 1977, Bird and colleagues randomized 42 inflammatory arthritis patients, mostly RA but some with psoriatic arthritis, to IA triamcinolone or IA methotrexate for knee synovitis [8]. The methotrexate dosing was variable. With thermographic index as the primary outcome, methotrexate was inferior to triamcinolone, in both the RA and psoriatic arthritis patients. They did not report on adverse effects. Hall and colleagues performed a randomized double blind trial in 15 RA patients, comparing IA MTX (given as three doses) with IA saline for knee synovitis [9]. All patients had arthroscopic saline washout at the onset, before trial medicine administration. At 3 month arthroscopic assessment, no significant difference between the two groups was found. They also studied 5 patients with psoriatic arthritis and noticed some short-term benefit from IA methotrexate in this group. They did not report on adverse effects. Both the aforementioned studies made the interesting observation of the injected MTX entering the contralateral non-injected knee within an hour [8, 9]. A larger study looked at 82 RA patients with persistent knee symptoms [10]. They had three intervention arms: IA triamcinolone, IA triamcinolone plus IA methotrexate, and IA triamcinolone plus IA rifampin. At 3 months, the rifampin group had better control, based on a pain-scale outcome, but at 6 months, all groups were similar. Two (7.0%) patients in the methotrexate group reported post-injection pain compared to 11 in the rifampin group. One (3.5%) methotrexate group patient developed mouth ulcers at day 10. The 2004 study by Hasso compared IA MTX plus triamcinolone (20 mg each) with IA triamcinolone (20 mg) alone in 38 patients with inflammatory knee synovitis [11]. 23 patients had RA and the rest had other seronegative arthritides. No meaningful difference was noted between the two groups at 24 weeks of follow-up. No local adverse side effects occurred in either group. The most recent trial investigating IA methotrexate was from 2018 [12]. This study enrolled 100 RA patients and was unique as it included elbows, wrists and ankles but no knees. One group received IA triamcinolone 40 mg once in the affected joint, while the other group received weekly IA methotrexate at 10 mg per injection for 8 weeks. All injections were with ultrasound (US) guidance. At 20 weeks, they found IA MTX was superior to IA triamcinolone in terms of pain scores and US parameters (both gray-scale and Doppler). In terms of adverse effects, two (3.5%) patients in the methotrexate group developed oral ulcers while one (1.8%) developed post-injection nausea. Three (6.9%) patients in the triamcinolone group reported post-injection arthritis flare.

### Intraarticular etanercept

Table 2 encompasses the findings of the intra-articular TNFi therapies. Bliddal et al. investigated single injections of IA etanercept 25 mg vs. IA methylprednisolone 40 mg under US guidance in 38 RA patients [13]. RA flare in a joint (elbow, wrist or knee) was treated, with wrists being the most common. At 4 weeks follow-up, there was no difference in pain outcome between the two groups. One patient (5.6%) in the TNF-i group developed a lower extremity rash while one patient (5.0%) in the steroid group developed atrial fibrillation. A 2008 study compared the effectiveness of single IA etanercept 25 mg vs. IA methylprednisolone 40 mg under US guidance in the wrist joints of 25 RA patients [14]. In addition to clinical outcomes, they also assessed response by US and magnetic resonance imaging (MRI). The outcomes in both groups were similar. Notably, clinical improvement was noted in both groups but, surprisingly, imaging improvement was absent; in fact, deterioration was noted. They did not report on adverse effects. Roux from France compared IA etanercept 25 mg vs. IA betamethasone 4 mg, given under radiographic guidance, in elbow, wrist (most common), and knee or ankle monoarthritis in 34 randomized RA patients [15]. Clinical outcomes were similar, with both groups showing significant improvement, at 4 and 24 weeks. One (5.9%) phlebitis occurrence was noted in the etanercept group. Aalbers et al. investigated 11 RA patients (9 knee joints), as part of a larger group of 30 patients (the rest with psoriatic arthritis), for efficacy of a single IA etanercept 25 mg vs. IA normal saline [16]. Etanercept led to a statistically significant improvement in composite outcomes at 2 weeks, with the pain response sustained till 6 weeks. Overall adverse events were not statistically significant between the two groups. The most recent study investigating etanercept was from 2020 [18]. 50 RA patients with active monoarthritis were randomized to either a single IA etanercept (25 mg for wrist and ankle joints and 50 mg for knee joints) vs. IA methylprednisolone 40 mg in all joints, under US guidance. At week 1, etanercept was better; at week 4, both were equal; and at week 12, methylprednisolone was better based on clinical assessment outcomes. US results were mixed. No serious adverse effects occurred.

**Table 2:** Randomized Control Trials Evaluating Intraarticular TNFi for Persistent Monoarthritis in RA

Author, Year, Country	N of RA	Objective (s)	Study Design	Time in Weeks	Primary Outcome	Joints	Results*	Conclusions	Adverse Events
Bliddal [13], 2006, Denmark	38 TNFi: 18 Steroid: 21	Comparison of IA etanercept vs methylprednisolone	Randomized, Double-blind	4	VAS	Elbow, wrist and knee	Within-group analysis suggested that methylprednisolone trended towards stronger improvements from baseline though there was no statistical difference between VAS scores in the two groups	No statistical difference between methylprednisolone and etanercept, though methylprednisolone had a larger within-group benefit	1 patient developed atrial fibrillation after steroid injection, 1 patient in the Etanercept group developed a skin eruption lasting 2 months
Boesen [14], 2008, Denmark	25 TNFi: 13 Steroid: 12	Comparison of IA etanercept vs methylprednisolone	Randomized, Double-blind	4	MRI and US findings, swollen target joint score, tender target joint score, physician VAS	Wrist	Clinical measures of swollen target joint score, tender target joint score, and VAS improved over 4 weeks though there was no statistical difference between the groups; no difference between groups on imaging outcomes	IA etanercept was not superior to methylprednisolone on MRI or US-Doppler imaging	
Roux [15], 2011, France	34 TNFi: 17 Steroid: 17	Comparison of IA etanercept vs corticosteroid injections	Randomized, Double-blind	24	Target joint pain	Elbow, wrist, knee, and ankle	No statistical difference in VAS or HAQ scores between the 2 groups, though they both showed improvement from baseline	Both groups showed improvement without significant difference between the treatments	1 episode of phlebitis in the Etanercept group
Aalbers [16], 2015, The Netherlands	30, 11 with RA TNFi: 22 Placebo: 9	Comparison of IA etanercept vs placebo	Randomized, Double-blind	6	Composite change index based on VAS, clinical assessments, joint swelling + functional disability, patient global assessment and provider global assessment	MCP, knee and ankle	Etanercept improved the composite change index statistically for the first 2 weeks after injection compared with placebo (*p* < 0.001)	IA etanercept appeared to be an effective strategy, at least transiently, with minimal side effects	Mild and transient flu-like symptoms and GI complaints occurred in 32% of Etanercept patients and 25% with placebo (*P* = 0.55)
Carubbi [17], 2016, Italy	82 total, 41 with RA TNFi: 20 Steroid: 21	Comparison of IA TNFi (Infliximab, Etanercept, or Adalimumab) vs corticosteroid	Randomized, Single-blind	52	VAS improvement and safety	Shoulder elbow, wrist, MCP, PIP, hip, knee	There was an improvement greater than 20% for jVAS of involved joint pain in patients injected with TNFi. In RA patients (*p* < 0.001 for all time points)	IA TNFi was well-tolerated and resulted in at least equal efficacy compared with IA steroids	Temporary soreness at the injection site
Salem [18], 2020, Egypt	50 TNFi: 25 Steroid: 25	Comparison of IA etanercept vs methylprednisolone	Randomized	12	DAS28, MHAQ functional assessment, VAS, lab and US findings	Knee, wrist, ankle, elbow	VAS and clinical scores improved in both groups. The etanercept group had a statistically greater improvement over steroids in week 1 VAS and tenderness score (*p* = 0.007, *p* = 0.008 respectively); this reversed, and the steroid group was significantly more improved than the etanercept group at 12 weeks (*p* = 0.001, 0.005 respectively)	Comparable improvements seen between the IA etanercept and methylprednisolone groups	Temporary localized pain in one etanercept patient

*DAS28* disease activity score 28; *GA* global assessment; *HAQ* health assessment questionnaire; *IA* intra-articular; *MCP* metacarpophalangeal; *MHAQ* modified health assessment questionnaire; *MRI* magnetic resonance imaging; *PIP* proximal interphalangeal; *PsA* psoriatic arthritis; *RA* rheumatoid arthritis; *TNFi* tumor necrosis factor inhibitor; *US*:ultrasound; *VAS* visual analogue scale; *jVAS* joint visual analogue scale

*: When not given, *p*-value was not reported in the study or was statistically not significant

### Other intraarticular TNF-i

This Italian study had 41 RA patients [17]. They compared IA TNF-i (adalimumab 40 or etanercept 50 or infliximab 100) mg vs. IA triamcinolone 40 mg, each injected monthly under US guidance for a total of 3 doses. Wrists, metacarpophalangeal joints and knees were the most commonly involved joints. Interestingly, patients were also on systemic TNF-i therapy. The agent used for systemic therapy was utilized for intra-articular therapy if they were randomized to the TNFi treatment arm. Primary outcome was a visual pain scale, with imaging based secondary outcomes. Pain outcomes were significantly better in the TNF-i group up to 24 weeks of follow-up. Gray-scale and Doppler US as well as MRI improvements were significantly more pronounced in the TNF-i group. The response was better in the large joints. Clinical remission was achieved earlier in the TNF-i group (4 weeks) vs. the triamcinolone (8 weeks) group. The TNF-i group had no flares compared to several in the triamcinolone group at 52 weeks of follow-up. They noted that the TNF-i effect was independent of the type of TNF-i. No serious adverse events were reported.

### Risk of Bias assessment

Out of the 6 included trials that evaluated IA methotrexate, 4 of the studies were deemed at high risk of bias, 1 with some concern of bias, and 1 with low risk of bias (Table 3). In trials evaluating IA TNFi therapy, 1 study was deemed at high risk of bias, 1 with concern for bias, and 4 studies were considered at lower risk of bias (Table 4).

**Table 3:**
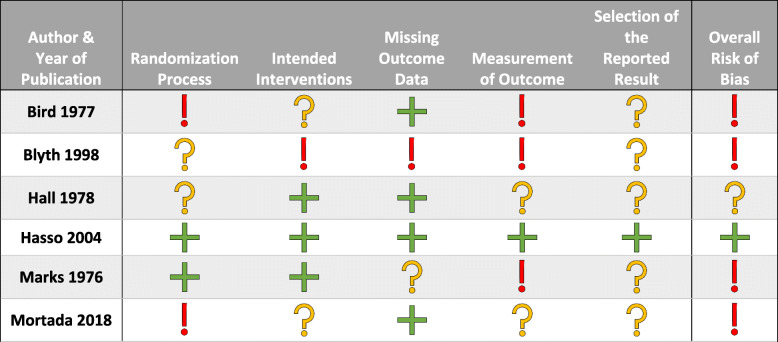
Revised Cochrane risk-of-bias tool for randomized trial for intra-articular methotrexate trials

Key:  = Low risk of bias  = Some concern of bias  = High risk of bias

**Table 4:**
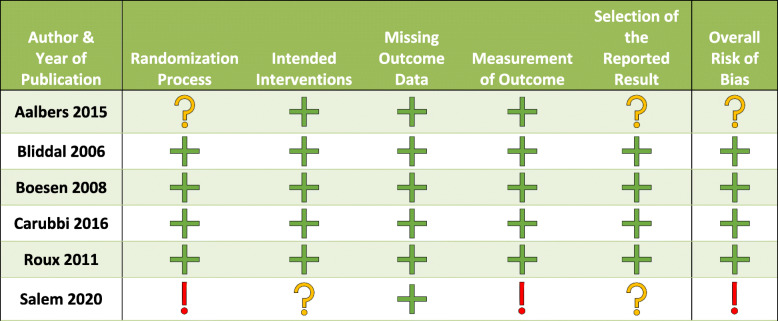
Revised Cochrane risk-of-bias tool for randomized trial for intra-articular TNFi trials

Key:  = Low risk of bias  = Some concern of bias  = High risk of bias

## Discussion

There is an unmet need of IA agents to control active inflammatory monoarthritis in patients with underlying inflammatory joint diseases, primarily RA, when IA corticosteroids fail to work, lose efficacy or cause adverse effects and systemic therapy is not the preferred option. MTX and etanercept have been most studied in this respect.

MTX has been studied as an IA agent either in combination with corticosteroids or as a substitute for corticosteroids in refractory monoarthritis. A 2004 Welsh study anecdotally mentioned that IA MTX and IA corticosteroid combination was being commonly used [11]. In our analysis, MTX was not found to be a useful treatment for mono-articular synovitis of the knee. Studies comparing IA MTX to IA saline and addition of IA MTX to other IA corticosteroids showed no benefit. What about using IA MTX in place of IA corticosteroids, if the latter are ineffective or contraindicated? The only study with a head-to-head equivalent comparison of IA MTX with IA corticosteroids found no benefit with MTX thus casting doubt on using IA MTX as an corticosteroid alternative [8]. The only study that favored IA MTX comparing it to IA corticosteroids, and evaluating non-knee joints, was the 2018 study comparing one IA triamcinolone injection to a total of eight IA MTX injections given weekly [12]. Notably, this was also the only study, in our review, that studied joints other than the knees (all other studies investigated the knee joints). However, any meaningful conclusion is difficult to ascertain because of this major difference in the administration frequency. Additionally, weekly IA MTX may not be clinically feasible. What we can extrapolate is that weekly IA injections of MTX do not have any substantial short-term adverse effects. In summary, there is no evidence in favor of using IA MTX for treatment of persistent knee synovitis. There may be some very weak evidence to support its use in non-knee monoarthritis.

IA corticosteroids has been an integral part of RA management. However, this may not remain the case in the future, necessitating alternate choices. In osteoarthritis, IA corticosteroid use is starting to fall out of favor due to lack of benefit and concerns for adverse effects. Some osteoarthritis studies have raised concerns about cartilage damage from IA corticosteroid exposure in addition to the lack of response [19]. The clinical relevance of such cartilage degradation, however, is unclear at the individual patient level [20]. While many new agents are under investigation as IA agents for OA, such momentum is lacking in RA.

One potential reason for this lack of benefit, especially from single IA MTX injections, is due to the elimination half-life of IA MTX which is just 2.9 h [21]. This time is insufficient for any cytotoxic effect to cause a chemical synovectomy. Therefore, there may be some rationale behind repeated IA injections of MTX and indeed the 2018 study in our analysis showed a response [12]. These pharmacodynamics have generated an interest in developing conjugated compounds to provide longer retention of IA MTX [22]. For reference, the half-life of IA triamcinolone acetonide is between 3.2 and 6.4 days [23]. Others have postulated that methotrexate does not have significant intracellular uptake with IA administration, and this could decrease the adenosine release hypothesized to be a major mechanism of action in rheumatoid arthritis [24, 25].

TNF is found in inflamed joints and thus a therapeutic response from an IA TNF-i injection is expected [26]. IA TNF-i can bind the local TNF to ameliorate inflammation and pain. It is possible that lack of TNF-i uptake in a joint may be the reason for persistent monoarthritis in an otherwise well-controlled RA patient on TNF-I therapy. This may be particularly true for the knee joint with its large synovial surface area and higher inflammatory milieu. IA etanercept studies included in our review were more helpful, compared to the IA MTX studies, as they all compared etanercept with IA corticosteroids or saline. No head-to-head IA corticosteroid study showed inferiority of IA etanercept [13–15]. Thus, IA etanercept could potentially be used in lieu of an IA corticosteroid, if needed. More importantly, and unlike IA MTX, IA etanercept was superior when compared to normal saline at 2 weeks [16]. This 2 week response duration is consistent with the half-life of etanercept, which is 68 h based on subcutaneous administration as IA half-life is unknown [27]. Rapid joint clearance, mediated by an efficient lymphatic drainage system, remains a challenge for IA therapies. Another study also showed the superiority of etanercept compared to methlyprednisone at 1 week, equality at 4 weeks and superiority of methlyprednisone at 12 weeks [18]. The important question of the utility of serial IA etanercept injections, like every 3 months similar to corticosteroids, has not been investigated. The study by Roux et al. showed sustained similarity in response between IA etanercept and IA betamethasone till 24 weeks, which is encouraging [15]. The study by Carubbi investigated three IA TNF-i (adalimumab, etanercept and infliximab) compared to IA corticosteroids and found IA TNF-i to generate significantly superior clinical and imaging outcomes [17]. Reassuringly, they also did not report any concerning adverse effects especially considering patients were also on parenteral TNF-i (similar to the IA agent). None of the included studies in our review investigated combined IA corticosteroid and TNF-i. However, a case series showed sustained 12 months remission when the active joint with synovitis was injected with such a combination [28]. Adalimumab (40 mg) was the main TNF-i (four out of five patients) used in this series. It is notable, that the benefit was only seen in TNF-i naïve patients. An uncontrolled study has shown infliximab as an effective IA therapy, even in patients already taking a TNF-i [29]. Ultrasound findings did show an improvement in this study. On the contrary, some infliximab series have not shown any benefit [30]. There is some basis to suggest efficacy from a second IA TNF-i injection as the first one may help reduce vascularity thus limiting systemic absorption and promoting a longer local effect from the subsequent injection [1, 31].

Overall, the IA therapy with MTX and TNF-i was well-tolerated with no notable adverse effects in the included studies. US usage ensured proper drug placement and allayed concerns of IA reactions from TNF-i placement [32]. We do note a case of development of miliary tuberculosis after an IA TNF-i injection [33]. Therefore, infection screening process prior to IA TNF therapy should be similar to that of systemic therapy. Anti-drug antibody formation after a single IA TNF-i has been reported [34].

Our study appears to be the first systematic review investigating the utility of non-corticosteroid immunosuppressive IA therapies for persistent monoarthritis in inflammatory arthritis patients. Considering the study question, publication bias risk is possible but less likely because even a negative result should not preclude publication. Limitations of this systematic review include the exclusion of non-English language studies which may create bias, variability in treatment doses, and heterogeneity of study protocols. Since these studies assessed intra-articular therapies, a strong component of placebo therapeutic response is possible and cannot be discounted [35]. Most of the studies investigating TNF-i had low risk of bias while MTX studies generally had higher bias risk. No cost-effectiveness analyses were reported.

## Conclusion

In conclusion, MTX likely has minimal utility, if any, as an IA agent for treatment of monoarthritis in patients with inflammatory arthritis. TNF-i in general, and specifically etanercept, have data supporting their use as IA agents in select patients with inflammatory arthritis when systemic treatment is not an option and IA corticosteroids cannot be used. More research is needed to investigate the optimal dose and frequency of IA TNF-i as well as to investigate the long-term results of IA TNF-i therapy. Cost-effectiveness data is also needed. Development of novel IA agents will greatly facilitate treatment of inflammatory monoarthritis.

## Supplementary Information

**Additional file 1.** Supplementary Table 1: Sample search strategy.)

## Data Availability

The data utilized for this article can be found individually through the articles assessed, or the dataset can be requested from the authors.

## References

[1] Fisher BA, Keat A (2006). Should we be using intraarticular tumor necrosis factor blockade in inflammatory monoarthritis?. J Rheumatol.

[2] Weitoft T, Uddenfeldt P (2000). Importance of synovial fluid aspiration when injecting intra-articular corticosteroids. Ann Rheum Dis.

[3] Kim SJ, Jung KA (2007). Arthroscopic synovectomy in rheumatoid arthritis of wrist. Clin Med Res.

[4] Amini A, Yahyanezhad S, Velez E, Gholamrezanezhad A, Fotoohi M, Jafari E, Assadi M (2020). Prospective evaluation of phosphorus-32 radiation synovectomy in patients with severe and chronic rheumatoid arthritis unresponsive to conventional medical treatment. Nucl Med Commun.

[5] Liberati A, Altman DG, Tetzlaff J, Mulrow C, Gotzsche PC, Ioannidis JP (2009). The PRISMA statement for reporting systematic reviews and meta-analyses of studies that evaluate healthcare interventions: explanation and elaboration. BMJ..

[6] Sterne JAC, Savovic J, Page MJ, Elbers RG, Blencowe NS, Boutron I (2019). RoB 2: a revised tool for assessing risk of bias in randomised trials. BMJ..

[7] Marks JS, Stewart IM, Hunter JA (1976). Intra-articular methotrexate in rheumatoid arthritis. Lancet..

[8] Bird HA, Ring EF, Daniel R, Bacon PA (1977). Comparison of intra-articular methotrexate with intra-articular triamcinolone hexacetonide by thermography. Curr Med Res Opin.

[9] Hall GH, Jones BJ, Head AC, Jones VE (1978). Intra-articular methotrexate. Clinical and laboratory study in rheumatoid and psoriatic arthritis. Ann Rheum Dis.

[10] Blyth T, Stirling A, Coote J, Land D, Hunter JA (1998). Injection of the rheumatoid knee: does intra-articular methotrexate or rifampicin add to the benefits of triamcinolone hexacetonide?. Br J Rheumatol.

[11] Hasso N, Maddison PJ, Breslin A (2004). Intra-articular methotrexate in knee synovitis. Rheumatology (Oxford).

[12] Mortada MA, Abdelwhab SM, Elgawish MH (2018). Intra-articular methotrexate versus corticosteroid injections in medium-sized joints of rheumatoid arthritis patients-an intervention study. Clin Rheumatol.

[13] Bliddal H, Terslev L, Qvistgaard E, Konig M, Holm CC, Rogind H, Boesen M, Danneskiold-Samsøe B, Torp-Pedersen S (2006). A randomized, controlled study of a single intra-articular injection of etanercept or glucocorticosteroids in patients with rheumatoid arthritis. Scand J Rheumatol.

[14] Boesen M, Boesen L, Jensen KE, Cimmino MA, Torp-Pedersen S, Terslev L, Koenig M, Danneskiold-Samsøe B, Røgind H, Bliddal H (2008). Clinical outcome and imaging changes after intraarticular (IA) application of etanercept or methylprednisolone in rheumatoid arthritis: magnetic resonance imaging and ultrasound-Doppler show no effect of IA injections in the wrist after 4 weeks. J Rheumatol.

[15] Roux CH, Breuil V, Valerio L, Amoretti N, Brocq O, Albert C (2011). Etanercept compared to intraarticular corticosteroid injection in rheumatoid arthritis: double-blind, randomized pilot study. J Rheumatol.

[16] Aalbers C, Gerlag D, Vos K, Vervoordeldonk M, Landewe R, Tak PP (2015). Intra-articular etanercept treatment in inflammatory arthritis: a randomized double-blind placebo-controlled proof of mechanism clinical trial validating TNF as a potential therapeutic target for local treatment. Joint Bone Spine..

[17] Carubbi F, Zugaro L, Cipriani P, Conchiglia A, Gregori L, Danniballe C, Letizia Pistoia M, Liakouli V, Ruscitti P, Ciccia F, Triolo G, Masciocchi C, Giacomelli R (2016). Safety and efficacy of intra-articular anti-tumor necrosis factor alpha agents compared to corticosteroids in a treat-to-target strategy in patients with inflammatory arthritis and monoarthritis flare. Int J Immunopathol Pharmacol.

[18] Salem RM, El-Deeb AE, Elsergany M, Elsaadany H, El-Khouly R. Intra-articular injection of etanercept versus glucocorticoids in rheumatoid arthritis patients. Clin Rheumatol. 2020;40(2), 557–564. 10.1007/s10067-020-05235-9. 32623650

[19] McAlindon TE, LaValley MP, Harvey WF, Price LL, Driban JB, Zhang M (2017). Effect of intra-articular triamcinolone vs saline on knee cartilage volume and pain in patients with knee osteoarthritis: a randomized clinical trial. JAMA..

[20] Bacon K, LaValley MP, Jafarzadeh SR, Felson D (2020). Does cartilage loss cause pain in osteoarthritis and if so, how much?. Ann Rheum Dis.

[21] Wigginton SM, Chu BC, Weisman MH, Howell SB (1980). Methotrexate pharmacokinetics after intraarticular injection in patients with rheumatoid arthritis. Arthritis Rheum.

[22] Boechat AL, de Oliveira CP, Tarrago AM, da Costa AG, Malheiro A, Guterres SS (2015). Methotrexate-loaded lipid-core nanocapsules are highly effective in the control of inflammation in synovial cells and a chronic arthritis model. Int J Nanomedicine.

[23] Derendorf H, Mollmann H, Gruner A, Haack D, Gyselby G (1986). Pharmacokinetics and pharmacodynamics of glucocorticoid suspensions after intra-articular administration. Clin Pharmacol Ther.

[24] Tada M, Inui K, Okano T, Mamoto K, Koike T, Nakamura H (2019). Safety of intra-articular methotrexate injection with and without electroporation for inflammatory small joints in patients with rheumatoid arthritis. Clin Med Insights Arthritis Musculoskelet Disord.

[25] Maksimovic V, Pavlovic-Popovic Z, Vukmirovic S, Cvejic J, Mooranian A, Al-Salami H (2020). Molecular mechanism of action and pharmacokinetic properties of methotrexate. Mol Biol Rep.

[26] Deleuran BW, Chu CQ, Field M, Brennan FM, Mitchell T, Feldmann M, Maini RN (1992). Localization of tumor necrosis factor receptors in the synovial tissue and cartilage-pannus junction in patients with rheumatoid arthritis. Implications for local actions of tumor necrosis factor alpha. Arthritis Rheum.

[27] Korth-Bradley JM, Rubin AS, Hanna RK, Simcoe DK, Lebsack ME (2000). The pharmacokinetics of etanercept in healthy volunteers. Ann Pharmacother.

[28] Haroon M, O'Gradaigh D (2010). Efficacy and safety of combining intra-articular methylprednisolone and anti-TNF agent to achieve prolonged remission in patients with recurrent inflammatory monoarthritis. Joint Bone Spine.

[29] Conti F, Ceccarelli F, Priori R, Iagnocco A, Signore A, Valesini G (2008). Intra-articular infliximab in patients with rheumatoid arthritis and psoriatic arthritis with monoarthritis resistant to local glucocorticoids. Clinical efficacy extended to patients on systemic anti-tumour necrosis factor alpha. Ann Rheum Dis.

[30] Bokarewa M, Tarkowski A (2003). Local infusion of infliximab for the treatment of acute joint inflammation. Ann Rheum Dis.

[31] Taylor PC (2005). Serum vascular markers and vascular imaging in assessment of rheumatoid arthritis disease activity and response to therapy. Rheumatology (Oxford).

[32] Bliddal H, Terslev L, Qvistgaard E, Recke P, Holm CC, Danneskiold-Samsoe B (2006). Safety of intra-articular injection of etanercept in small-joint arthritis: an uncontrolled, pilot-study with independent imaging assessment. Joint Bone Spine..

[33] Li SG (2011). Clinical image: development of miliary tuberculosis following one intraarticular injection of etanercept. Arthritis Rheum.

[34] Zufferey P, Perreau M, So A (2015). High level of anti-drug antibodies after intra-articular injection of anti-TNF. Rheumatology (Oxford).

[35] Previtali D, Merli G, Di Laura FG, Candrian C, Zaffagnini S, Filardo G (2020). The long-lasting effects of "placebo injections" in knee osteoarthritis: a Meta-analysis. Cartilage..

