# Supplementary material for: Intra-articular therapy with methotrexate or tumor necrosis factor inhibitors in rheumatoid arthritis: a systematic review
Source: BMC Musculoskelet Disord. 2021 Sep 15;22:792. doi: 10.1186/s12891-021-04651-5 (PMC8444402; doi:10.1186/s12891-021-04651-5)
Supplement: Supplementary file 1 — Additional file 1. Supplementary Table 1: Sample search strategy.) [file 12891_2021_4651_MOESM1_ESM.docx]

| **#** | **Searches** | **Results** |
| --- | --- | --- |
| 1 | rheumatoid arthritis.mp. or exp Arthritis, Rheumatoid/ | 148287 |
| 2 | exp Injections, Intra-Articular/ or intra-articular inject*.mp. | 9847 |
| 3  4 | 1 and 2  exp lmmunosuppressive Agents/ or immunosuppressive agent*.mp. | 1201  320160 |
| 5 | exp Antirheumatic Agents/ or antirheumatic agent*.mp. | 426932 |
| 6 | anti-rheumatic agent*.mp. | 161 |
| 7 | 5 or 6 | 426981 |
| 8 | abatacept.mp or exp Abatacept | 3747 |
| 9 | exp Azathioprine/ or azathioprine.mp. | 14632 |
| 10 | belimumab.mp. | 683 |
| 11 | exp Certolizumab Pegol/ or certolizumab.mp. | 1298 |
| 12 | cyclophosphamide.mp. or exp Cyclophosphamide/ | 74012 |
| 13 | cyclosporine.mp. or exp Cyclosporine/ | 45930 |
| 14 | etanercept.mp. or exp Etanercept | 8607 |
| 15 | leflunomide.mp. or exp Leflunomide/ | 2490 |
| 16 | methotrexate.mp. or exp Methotrexate/ | 54431 |
| 17 | sirolimus.mp. or exp Sirolimus/ | 22534 |
| 18 | adalimumab.mp. or exp Adalimumab/ | 8539 |
| 19 | enbrel.mp. | 318 |
| 20 | infliximab.mp. or exp Infliximab/ | 14997 |
| 21 | 4 or 7 or 8 or 9 or 10 or 11 or 12 or 13 or 14 or 15 or 16 or 17 or 18 or 19 or 20 | 654720 |
| 22 | 3 and 21 | 362 |
| 23 | limit 22 to english language | 298 |

Supplementary Table 1: Sample search strategy

Database(s): Ovid MEDLINE(R) 1946 to Present and Epub Ahead of Print, In-Process & Other Non-Indexed Citations and Ovid MEDLINE(R) Daily

-search ran 8/10/2020
